# Queer Definitions in Hospital Diet

**Published:** 1911-09-30

**Authors:** 


### QUEER DEFINITIONS IN HOSPITAL DIET.

Mr. A. Griffiths, the Secretary to the East Suffolk Hospital, Ipswich, who was asked by us to state the practice at his hospital, and give his views on this subject, writes as follows :—

Tea, sugar, and butter are included in the patients' dietary here, and are looked upon by Board and officials alike as necessities rather than luxuries. Surely the trouble to which patients would be put in having to provide these necessary articles of food for themselves—apart from the questions of the unsatisfactory articles they are likely to provide themselves with, or the extra work entailed upon the nursing staff—would far outweigh the question of cost to the institution, for this must be infinitesimally small when compared with the general outlay for provisions. The deletion of these items from the patients' bill of fare when they are included in that of the most menial servitor in the hospital would appear to be unreasonable. The cost of these little items is as nothing compared with the comfort they afford to the sufferers, and their inclusion in the free menu must help to make hospital life more endurable, whilst a regulation for their exclusion would, I am sure, be looked upon more in the light of a vexatious piece of red tape, than as an act of economy. Perhaps the conditions are different in the provinces from those in the Metropolis, but I submit that the above remarks would apply equally to all classes of patients treated here, and I fail to see how the question of the social position of the patient materially affects the argument.

